# Citric Acid-Treated PEDOT:PSS with Optimized Interfacial Energetics for Phosphorescent OLEDs Achieving over 20% EQE and Extended Lifetime

**DOI:** 10.3390/polym18091104

**Published:** 2026-04-30

**Authors:** Ming Wu, Wenqing Zhu, Zhiyin Feng, Qidi Lin, Lu Huang

**Affiliations:** 1 School of Material Science and Engineering, Shanghai University, 99 Shangda Road, Shanghai 200444, China; mingwu@shu.edu.cn (M.W.); iinmutsu@shu.edu.cn (Z.F.); qdlin@shu.edu.cn (Q.L.); lhuang@shu.edu.cn (L.H.); 2 Key Laboratory of Advanced Display and System Applications, Ministry of Education, Shanghai University, Yanchang Road 149, Shanghai 200072, China

**Keywords:** organic light-emitting diodes (OLEDs), PEDOT:PSS, citric acid, hole injection layer, carrier balance, interfacial engineering, conductive polymers

## Abstract

The hole injection layer (HIL) plays a critical role in achieving high efficiency and operational stability in organic light-emitting diodes (OLEDs). As a commonly used HIL, poly(3,4-ethylenedioxythiophene):poly(styrene sulfonate) (PEDOT:PSS) is limited by its intrinsically low electrical conductivity and mismatched work function alignment with the hole transport layer (HTL), leading to inefficient hole injection and carrier imbalance. In this work, a mild citric acid (CA) treatment is used to simultaneously enhance the conductivity of PEDOT:PSS through the partial removal of insulating PSS and tune its work function for improved energy level alignment at the anode interface. This simultaneous optimization effectively enhances the hole transport capability, successfully matching the electron transport capability to realize highly improved charge carrier balance within the device. Consequently, Ir(ppy)_3_-based phosphorescent OLEDs featuring the optimally treated PEDOT:PSS HIL deliver a maximum external quantum efficiency of 20.37%, representing a 21% improvement over devices using pristine PEDOT:PSS, along with a twofold extension in operational lifetime. This strategy demonstrates a simple and controllable approach to interfacial engineering, providing practical guidance for the development of high-performance and stable OLEDs.

## 1. Introduction

Organic light-emitting diodes (OLEDs) are now widely used in commercial displays and solid-state lighting due to their wide color gamut and high contrast ratio [1,2,3,4].

For practical applications, realizing high efficiency and a long operational lifetime remains the ultimate goal for OLED technology [5,6]. Device performance is often critically limited by charge injection imbalance and interfacial degradation at the anode side. Inefficient hole supply disrupts the carrier balance within the emissive layer, limiting the overall device efficiency [7,8]. Therefore, interface engineering through rational modification of the anode/hole injection layer (HIL) junction is essential to enhance hole injection and suppress interfacial degradation [9,10].

The HIL plays a key role in determining overall device performance. It serves as an intermediate buffer layer to reduce the hole injection barrier and suppresses leakage current. Among various HIL materials, including transition metal oxides (e.g., MoO_3_ [11], WO_3_ [12], VO_x_ [13], NiO_x_ [14]) and small molecules (e.g., HAT-CN [5]), poly(3,4-ethylenedioxythiophene):poly(styrene sulfonate) (PEDOT:PSS) remains the most widely used in solution-processed OLEDs due to its excellent solution processability and high visible light transmittance.

However, pristine PEDOT:PSS suffers from intrinsically low electrical conductivity (typically around 1 S cm^−1^), a work function (approx. 5.2 eV) that exhibits sub-optimal energetic alignment with the adjacent layers (i.e., the ITO anode and common hole transport layers), and strong acidity combined with hygroscopicity that accelerates ITO corrosion and indium diffusion into the emissive zone. [15,16].

To address these limitations, various modification strategies have been extensively explored. Although incorporating high-boiling-point solvents (e.g., DMSO [17,18,19,20], EG [21,22]) into PEDOT:PSS facilitates phase separation and enhances electrical conductivity, this approach leaves the intrinsic acidity of PSS unresolved, leading to persistent ITO corrosion during long-term operation. Alternatively, alkaline solutions (e.g., NaOH [23,24]) can effectively suppress interfacial acidity; however, they induce substantial de-doping and disrupt crystalline PEDOT domains, which drastically deteriorates the electrical conductivity. Furthermore, adding functional polymers (e.g., PSSA [25]), organic modifiers (e.g., HQ [26]), or inorganic salts (e.g., potassium sulfate [27,28]) can tailor the work function and improve specific optoelectronic properties. Nevertheless, an excessive concentration of mobile ions or insulating components compromises charge transport in the device, and the operational stability of these treated devices is rarely reported. Lastly, post-treatment with strong mineral acids (e.g., H_2_SO_4_ [29]) can significantly boost conductivity by washing away insulating PSS. However, their highly corrosive nature poses severe environmental and safety concerns. Moreover, strong acids frequently shift the work function to values too shallow for efficient hole injection. Although weak-acid treatments of PEDOT:PSS have been reported previously, those studies were mainly developed to improve conductivity or enable flexible/ITO-free electrodes in photovoltaic devices [30,31,32,33]. In such systems, the primary emphasis is often placed on charge extraction and film conductivity. In contrast, for phosphorescent OLEDs, the hole-injection layer must simultaneously satisfy several more tightly coupled requirements, including adequate conductivity, proper energetic alignment with the hole-transport layer, low interfacial acidity, and compatibility with the corrosion-sensitive ITO anode. Therefore, the role of a mild acid treatment in OLEDs cannot be understood solely as a generic “PSS removal” process and requires a device-specific interfacial analysis.

In this work, citric acid (CA), a mild, biodegradable, and environmentally benign organic acid, is introduced as an effective alternative to harsh mineral acids. CA-treated PEDOT:PSS is employed as the HIL to enhance the electroluminescent performance of Ir(ppy)_3_-based phosphorescent OLEDs. The CA treatment selectively removes the excess insulating and acidic PSS from the film matrix, thereby reducing the intrinsic acidity. Under the optimized condition (41 wt.% CA), this processing simultaneously improves the electrical conductivity of the HIL and tunes its work function for better energy level alignment. Distinct from previous reports focusing primarily on conductivity, this work provides direct XPS depth profiling evidence of suppressed indium diffusion, linking surface reconstruction to interfacial stabilization. While state-of-the-art Ir(ppy)_3_-based green phosphorescent OLEDs using complex multilayer architectures have achieved excellent efficiencies [34,35], the EQE of 20.37% obtained here with a simplified solution-processed HIL remains meaningful, especially with improved stability. More importantly, the device demonstrates a twofold extension in lifetime compared to the pristine reference. This work demonstrates that tailoring the interfacial energetics and chemical stability of PEDOT:PSS via an eco-friendly route is an effective strategy, providing a reliable pathway for designing high-performance solution-processable OLED products.

## 2. Materials and Methods

Materials: Citric acid (99.5% purity) was purchased from Aladdin, Shanghai, China. Poly(3,4-ethylenedioxythiophene):poly(styrenesulfonate) (PEDOT:PSS) aqueous dispersion (Clevios™ P VP AI 4083) was acquired from Heraeus, Hanau, Germany. The organic functional materials, including 4,4′-Cyclohexylidenebis[N,N-bis(4-methylphenyl)benzenamine] (TAPC, 99.5%), 1,3-bis(N-carbazolyl)benzene (mCP, 99%), Tris[2-(p-tolyl)pyridine]iridium(_III_) (Ir(ppy)_3_, 99%), 2,2′,2″-(1,3,5-Benzinetriyl)-tris(1-phenyl-1-H-benzimidazole) (TPBi, 99.8%), and 8-hydroxyquinolinato lithium (Liq, 99.9%), were purchased from Luminescence Technology Corp. (Lumtec), New Taipei City, Taiwan. All materials were used as received without further purification. The OLED device architecture and molecular structures of the key materials are provided in Figures S1 and S2, Supplementary Materials.

Preparation of CA Solutions and Film Fabrication: To systematically investigate the mechanism of surface reconstruction under varying acidic strengths, citric acid aqueous solutions were prepared with mass fractions of 24, 41, and 58 wt. %, corresponding to mild, intermediate, and near-saturation regimes, respectively. Specific amounts of anhydrous CA powder were dissolved in deionized (DI) water to achieve the target mass concentrations. For brevity, the treated films are hereafter denoted as CA-24, CA-41, and CA-58, respectively. The OLED devices were fabricated on patterned ITO-coated glass substrates with a sheet resistance of 15 Ω sq^−1^. Prior to use, the substrates were sequentially ultrasonicated in detergent, DI water, acetone, and isopropanol, followed by a 15 min UV-ozone treatment to decompose surface organic contaminants and improve wettability. HIL fabrication via selective surface reconstruction: The pristine PEDOT:PSS films were initially deposited by spin-coating the commercial dispersion onto the cleaned ITO substrates at 5000 rpm for 40 s, followed by annealing at 130 °C for 15 min in ambient air to remove residual moisture. Subsequently, the selective surface reconstruction was performed via a controlled swelling-diffusion process. The prepared CA solutions (CA-24, CA-41, and CA-58) were applied drop-wise onto the annealed PEDOT:PSS films to fully cover the surface and allowed to interact for 15 min. This interaction time was optimized to ensure sufficient infiltration of CA molecules into the polymeric matrix. The films were then thoroughly rinsed with copious amounts of DI water to remove the solubilized PSS chains and any residual acid. Finally, the reconstructed films were re-annealed at 130 °C for 15 min to consolidate the conductive network. Device Assembly: Following the HIL preparation, the substrates were transferred to a high-vacuum thermal evaporation chamber (base pressure ≈ 1 × 10^−4^ Torr). The organic functional layers and the metal cathode were deposited sequentially through shadow masks with the following architecture: ITO/HIL (pristine or CA-modified, ~30 nm) / TAPC (40 nm)/mCP: 8 wt. % Ir(ppy)_3_ (30 nm)/TPBi (40 nm)/Liq (1 nm)/Al (100 nm). It should be noted that the thickness of the CA-treated HILs varied slightly depending on the etching degree, and the specific thickness for each sample was measured by profilometry and used for SCLC calculations (Table S1, Supplementary Materials). The active area of each device was defined as 5 mm^2^ by the intersection of the ITO anode and the Al cathode.

Characterization: The current density–voltage–luminance (J–V–L) characteristics and electroluminescence (EL) spectra of the OLEDs were measured using a Keithley 2400 source meter (Keithley, Solon, OH, USA) coupled with a PR-650 Spectra Scan colorimeter (Photo Research, Syracuse, NY, USA). The film thickness and surface morphology were characterized using a surface profilometer (ET150, Kosaka, Japan) and an atomic force microscope (AFM, Dimension Edge, Bruker, Germany) in tapping mode, respectively. Optical transmittance spectra were recorded with a UV7600 UV-vis spectrophotometer (Shanghai Lengguang Technology Co., Ltd., Shanghai, China). The surface chemical composition and electronic energy levels were investigated using X-ray photoelectron spectroscopy (XPS) and ultraviolet photoelectron spectroscopy (UPS) on a Thermo Scientific Escalab 250Xi system (Thermo, Waltham, MA, USA), utilizing monochromatized Al Kα (1486.7 eV) and He Iα (21.22 eV) radiation sources, respectively.

## 3. Results and Discussion

### 3.1. Physicochemical Properties and Morphological Evolution of CA-Treated Films

**Figure 1:**
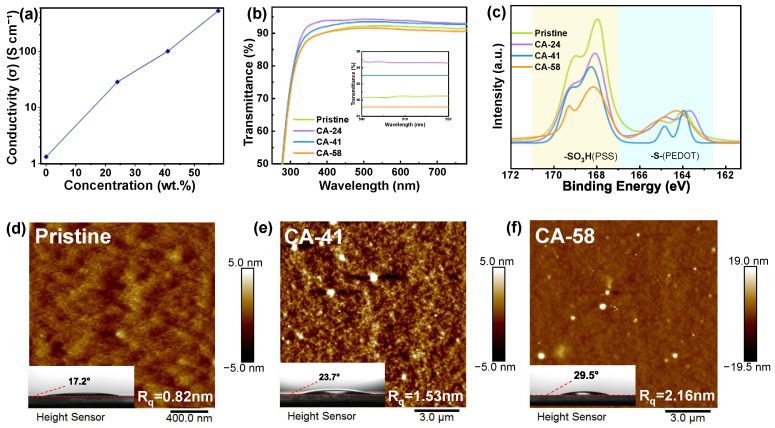
Fundamental optoelectronic properties and microscopic surface characterizations of the pristine and citric acid (CA) -treated PEDOT:PSS films. (**a**) Electrical conductivity as a function of CA concentration. (**b**) UV-Vis transmittance spectra, with the inset highlighting the detailed transmittance in the 500–520 nm range. (**c**) High-resolution XPS S 2p core-level spectra indicating the reduction in PSS. AFM topographic images of (**d**) pristine PEDOT:PSS, (**e**) CA-41-treated PEDOT:PSS, and (**f**) CA-58-treated PEDOT:PSS, showing the surface morphology evolution from a smooth and uniform film to a granular network and then to larger PEDOT-rich aggregates at high CA concentration. The insets in (**d**–**f**) display the corresponding WCA measurements.

To establish the viability of CA-treated PEDOT:PSS as a high-performance HIL, the fundamental optoelectronic properties were first systematically evaluated. Figure 1a demonstrates a profound, concentration-dependent surge in electrical conductivity upon CA treatment. The pristine film exhibits a highly restricted conductivity of 1.34 S cm^−1^, which is primarily dictated by the thick encapsulation of conductive PEDOT domains by the insulating PSS shell [36]. In striking contrast, the modified films manifest a significant enhancement spanning over two orders of magnitude, consistent with the well-known role of acid-induced PSS removal and PEDOT reorganization in boosting conductivity in PEDOT:PSS systems [37]. The conductivity escalates monotonically to 28.85, 101.83, and 534.62 S cm^−1^ for the samples treated with 24, 41, and 58 wt.% CA solutions (hereafter denoted as CA-24, CA-41, and CA-58), respectively. Concurrently, the optical transmittance, a critical parameter for maximizing the light extraction efficiency of OLEDs, is well-preserved and even optimized. As presented in the UV–Vis transmission spectra and the corresponding magnified inset (Figure 1b), the selective treatment yields a net gain in transparency across the visible spectrum, paralleling reports where removal of excess PSS enhances both conductivity and transparency in acid-treated PEDOT:PSS transparent electrodes [38]. Specifically, the CA-41 film achieves a superior transmittance exceeding 94% in the 500–520 nm range, effectively outperforming the pristine counterpart and meeting typical transparency requirements for high-performance optoelectronic electrodes [39]. This optical enhancement is structurally attributed to the removal of the excess PSS-rich phase boundaries, which significantly reduces parasitic light absorption and internal scattering, a mechanism widely observed in mineral- and weak-acid-treated PEDOT:PSS films [37,40]. However, a slight optical regression is observed for the near-saturation CA-58 film. This minor transmission loss likely stems from enhanced surface scattering induced by excessive phase separation and residual micro-crystallites formed under highly concentrated acid treatment, analogous to the morphology-driven transparency trade-offs seen at extreme acid doping levels in PEDOT:PSS electrodes [41].

To thoroughly unravel the microscopic chemical origin dictating this conductivity leap, X-ray photoelectron spectroscopy (XPS) was employed to interrogate the surface chemical evolution. Figure 1c presents the S 2p core-level spectra, featuring two distinct spin-split doublets. The lower binding energy emission (163–165 eV) originates from the sulfur atoms in the conductive PEDOT backbone, while the broader, higher energy counterpart (167–171 eV) is clearly ascribed to the sulfonate moieties (-SO_3_H and -SO_3_^−^) of the insulating PSS chains [42]. Visually, the relative intensity of the PSS peak undergoes a dramatic attenuation with increasing CA concentration. A semi-quantitative analysis based on the integrated peak areas provides compelling evidence: the PEDOT-to-PSS ratio increases monotonically from 0.20 in the pristine film to 0.34 in the CA-41 film, ultimately reaching 0.43 in the CA-58 sample. This compositional evolution strongly corroborates a “proton exchange” mechanism [43]. The abundant H+ ions provided by the triprotic citric acid rapidly associate with the negatively charged PSS anions to form neutral PSSH species. This neutralization effectively screens the strong Coulombic interactions that typically bind PSS to the PEDOT backbone [44,45]. Consequently, the liberated insulating PSSH chains undergo phase segregation and are subsequently washed away during the selective reconstruction process, establishing highly efficient, unhindered electrical percolation pathways within the bulk film [46].

To further describe the film morphology of the PEDOT:PSS hole-injection layers, Atomic Force Microscopy (AFM) was employed to visualize the surface evolution after CA treatment. The pristine PEDOT:PSS surface (Figure 1d) exhibits a featureless, highly uniform topography with a low root-mean-square roughness (R_q_) of 0.82 nm (measured over a 2 × 2 μm^2^ scan area), which is characteristic of a thick, amorphous PSS-rich top skin.

Following the selective reconstruction, the remaining PEDOT chains undergo phase segregation. To comprehensively capture these expanded, larger-scale aggregated domains, a broader scan area (12.5 × 12.5 μm^2^) was employed for the CA-treated films. Under the optimized condition, the CA-41 film (Figure 1e) transforms into a highly interconnected, granular network with a moderately increased R_q_ of 1.53 nm. This distinct textural evolution strongly implies a nanoscale conformational transition. As reported in previous studies, liberated from the confinement of bulky PSS counter-ions, the underlying PEDOT chains can relax from a randomly coiled benzoid structure into an extended, highly delocalized linear quinoid configuration [23,47]. This structural ordering not only fosters enhanced interchain π-π stacking to support the high bulk conductivity but also expands the effective interfacial contact area with the overlying hole transport layer [48]. This tailored nanotexture provides a higher density of hopping sites, which effectively facilitates dense and uniform hole injection while maintaining sufficient surface uniformity for ideal interfacial contact.

However, in the case of the CA-58 sample, the R_q_ further increases to 2.16 nm, accompanied by the emergence of larger PEDOT-rich domains. While this aggressive phase separation significantly enhances lateral conductivity by forming highly conductive pathways, it introduces vertical inhomogeneity. These enlarged aggregates, visible as protruding features in Figure 1f, suggest the formation of conducting filaments rather than a uniform bulk film [29]. Crucially, this increased roughness may also degrade the physical contact with the subsequent hole transport layer (HTL), contributing to the increased hole extraction barrier discussed in the energy level analysis. Thus, CA-41 represents the optimal balance, where conductivity is maximized without compromising the interfacial uniformity required for balanced carrier injection.

Finally, the macroscopic impact of this selective reconstruction on surface energetics was validated via water contact angle (WCA) measurements. The inherent hydrophilicity of the pristine film (WCA = 17.2°, inset of Figure 1d) originates directly from the hygroscopic nature of the highly concentrated surface PSS. Following the selective removal of these water-absorbing chains, the CA-41 film exposes the intrinsically more hydrophobic PEDOT-rich core, progressively shifting the WCA to 23.7° (inset of Figure 1e), with further increases observed at higher concentrations. While the surface remains hydrophilic, this distinct compositional shift physically confirms the success of the phase segregation and reduces the density of hygroscopic sulfonate groups. This modification potentially mitigates water uptake at the anode interface, which is contributing to the operational stability of the resulting OLED devices.

### 3.2. Interfacial Energetics Engineering and OLED Performance Mechanism

**Figure 2:**
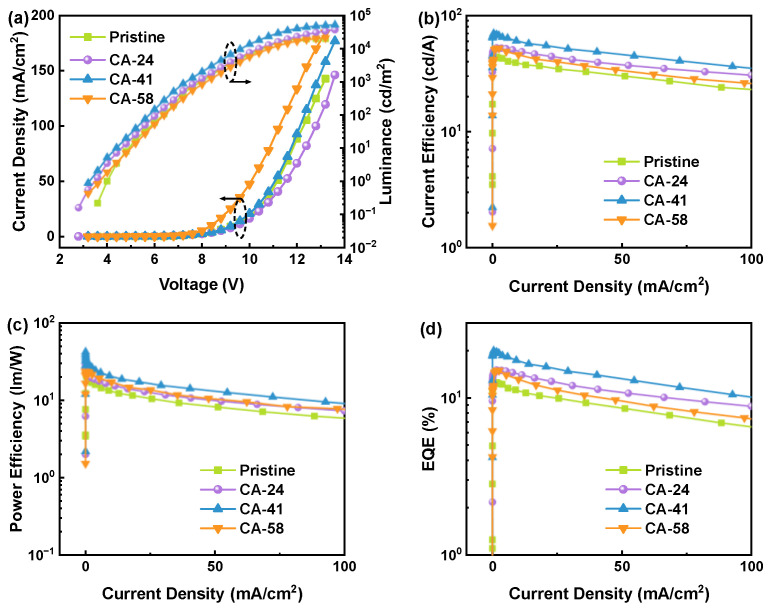
Electroluminescent characteristics of green OLEDs based on pristine and CA-treated PEDOT:PSS HILs: (**a**) J-V-L, (**b**) CE-J, (**c**) PE-J, (**d**) EQE-J.

To quantitatively evaluate the efficacy of the selective surface reconstruction on macroscopic device performance, Ir(ppy)_3_-based green phosphorescent OLEDs were fabricated, and their electroluminescent (EL) characteristics were systematically monitored. Figure 2 presents the current density–voltage–luminance (J-V-L) characteristics, current efficiency–current density (CE-J) characteristics, power efficiency–current density (PE-J) characteristics, and external quantum efficiency–current density (EQE-J) curves, while the key performance parameters are comprehensively summarized in Table 1.

As depicted in Figure 2a, all devices incorporating CA-treated PEDOT:PSS HILs exhibit a consistent and systematic reduction in driving voltage. For instance, the turn-on voltage (Von) is steadily suppressed from 3.76 V for the pristine device to 3.56 V for the CA-41 device, ultimately reaching a minimum of 3.53 V for the CA-58 device. This distinct downward trend is highly consistent with the substantial enhancement in the bulk electrical conductivity of the HILs observed in Figure 1, confirming that the effective removal of the insulating PSS shell significantly minimizes ohmic losses across the anode interface [49].

Regarding the overall device efficiency (Figure 2b–d), a prominent non-monotonic, “volcano-shaped” dependence on the CA treatment concentration is observed. While the introduction of 24 wt.% and 41 wt.% CA results in a progressive enhancement, the CA-41 device emerges as the optimal configuration. It achieves an exceptional maximum current efficiency (CE) of 70.20 cd A^−1^, a power efficiency (PE) of 42.18 lm W^−1^, and a peak external quantum efficiency (EQE) of 20.37%. These metrics represent a remarkable ~21% relative improvement in EQE compared to the pristine control device (16.83%). This breakthrough performance unambiguously demonstrates that the CA-41 treatment successfully optimizes the interfacial charge injection capabilities without introducing adverse non-radiative recombination pathways.

However, a clear performance trade-off is observed in the near-saturation treatment regime (CA-58). Although the CA-58 device exhibits the lowest turn-on voltage (3.53 V) and the highest lateral conductivity (534.62 S cm^−1^), its overall emission performance is lower than that of the optimized CA-41 device. The maximum CE decreases to 54.79 cd A^−1^, which is slightly lower than that of the pristine reference. This result indicates that high lateral conductivity alone is not sufficient to ensure efficient OLED operation. A stronger CA treatment likely induces excessive surface reconstruction, leading to an over-shifted work function and less favorable energy-level alignment at the hole-injection/hole-transport interface. Meanwhile, the rougher and more aggregated surface morphology under this condition may increase local interfacial inhomogeneity and reduce the uniformity of charge injection. These effects together provide a reasonable explanation for the lower overall efficiency of CA-58 [49].

**Figure 3:**
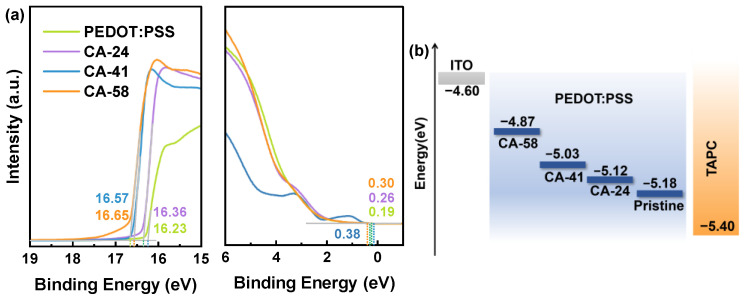
Interfacial energetics and energy level alignment characterizations. (**a**) Ultraviolet photoelectron spectroscopy (UPS) spectra showing the secondary electron cut-off regions (**left**) and the valence band onset regions (**right**) for the pristine and CA-treated PEDOT:PSS films. (**b**) Schematic energy level diagram illustrating the work function evolution of the HILs and the established step-wise energy cascade relative to the ITO anode and TAPC hole transport layer. The dashed and dotted guide lines in (**a**) indicate the linear extrapolations used to determine the secondary electron cut-off and valence band onset.

To systematically decipher the physical mechanisms underlying the breakthrough efficiency enhancement in CA-41 and the subsequent dramatic decline in the highly conductive CA-58 device, the evolution of the film energy levels was interrogated via Ultraviolet Photoelectron Spectroscopy (UPS). As depicted in the UPS spectra (Figure 3a), the secondary electron cut-off region exhibits a distinct and monotonic shift towards higher binding energies with increasing CA concentration. This systematic spectral shift directly translates to a monotonic reduction in the work function of the PEDOT:PSS films, as calculated and illustrated in the schematic energy level diagram (Figure 3b). Specifically, the work function decreases from 5.18 eV (pristine) to 5.12 eV (CA-24), 5.03 eV (CA-41), and ultimately to 4.87 eV (CA-58). This downward shift is not only a direct consequence of the selective removal of the high-ionization-potential PSS skin, but could also be attributed to the formation of a weak surface dipole layer. While absolute work function values may carry minor experimental uncertainty associated with secondary-electron cut-off determination, the observed trend is highly reproducible, and the relative differences are sufficient to support our discussion on interfacial energetic modulation. This downward shift stems primarily from the selective removal of the high-ionization-potential PSS skin. Given the high aqueous solubility of citric acid and the thorough rinsing protocol, significant CA residue on the film surface is unlikely. This assumption is consistent with the enhanced operational stability, as residual acid would otherwise compromise the ITO interface. Therefore, the work function evolution is attributed to the CA-induced selective removal and rearrangement of PSS at the outermost region, which reconstructs the local surface composition and interfacial dipole.

By comparing these precisely measured work functions with the highest occupied molecular orbital (HOMO) level of the TAPC hole transport layer (5.40 eV) and the work function of the ITO anode (approx. 4.60 eV), we completely mapped the interfacial energy landscape of hole injection. In the pristine device, the inherently high work function (5.18 eV) creates a substantial hole injection barrier of approx. 0.58 eV at the ITO/HIL interface. This barrier acts as a primary “intake bottleneck,” which severely restricts the efficient extraction of holes from the anode and inevitably leads to the high-voltage operation previously observed [50].

In comparison, the optimized CA-41 treatment delicately modulates the work function to establish a perfectly balanced, step-wise energy cascade where the energy barrier is evenly distributed between the anode interface (approx. 0.43 eV) and the organic interface (approx. 0.37 eV). Such a graded energetic alignment facilitates a remarkably smooth and barrier-free carrier relay, ensuring that the hole injection efficiency is effectively maximized across the entire anode-side junction.

In the case of the near-saturation CA-58 device, the aggressive acid treatment excessively reduces the work function to 4.87 eV. Although this minimizes the contact barrier with the ITO anode (approx. 0.27 eV), it simultaneously increases the energy barrier for hole extraction into the HTL (approx. 0.53 eV). This energetic mismatch, combined with the severe PEDOT aggregation discussed above, can partly offset the benefit of the high lateral conductivity and account for the lower efficiency of the CA-58 device [51].

**Figure 4:**
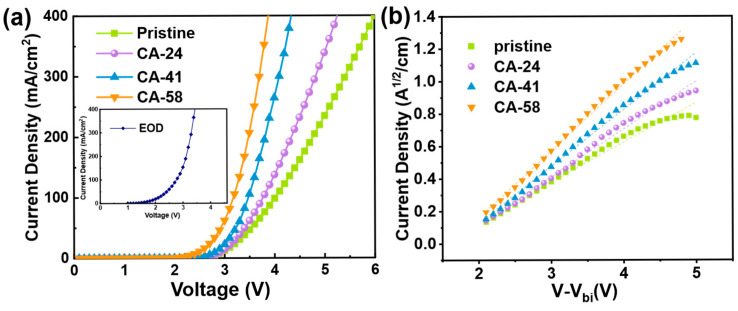
Charge transport properties and carrier balance analysis. (**a**) J-V curves of HODs based on pristine and CA-modified PEDOT:PSS films, compared with the EOD. (**b**) Square root of current density (J^1/2^) versus voltage (V − V_bi_) plots used for extracting zero-field mobilities. The dashed lines in (**b**) represent linear fits to the high-voltage region used for extracting the zero-field mobilities.

To further evaluate the macroscopic charge transport capability, single-carrier devices—specifically, hole-only devices (HODs) and electron-only devices (EODs)—were fabricated. As shown in Figure 4a, the EOD exhibits a substantially high current density, suggesting that electron injection and transport within this device structure are inherently efficient. By contrast, the HOD based on pristine PEDOT:PSS operates in a severe “hole-deficient” regime.

For a precise determination of this intrinsic charge transport capability, the zero-field mobility (μ_0_) was extracted using the space-charge-limited current (SCLC) model. Considering the built-in voltage (V_bi_) across the single-carrier devices, the trap-free SCLC regime at higher driving voltages is described by the modified Mott–Gurney equation:

J = (9/8) ε_0_ ε_r_ μ_h_ (V − V_bi_)^2^/L^3^

where J is the current density, ε_0_ is the vacuum permittivity, ε_r_ is the relative permittivity of the organic layer (assumed to be 3.0), μ is the carrier mobility, V is the applied voltage, V_bi_ is the built-in voltage resulting from the difference in work functions between the anode and cathode (In this device structure, 0.3 V is used), and L is the film thickness. Importantly, because the selective CA treatment reduces the film thickness, the specific measured thickness (L) of each corresponding film obtained via profilometry (Table S1, Supplementary Materials) was utilized for accurate calculation. By plotting J^1/2^ versus V (Figure 4b), the mobilities were reliably extracted from the slopes of the linear fits in the high-voltage region.

Based on this model, the extracted electron mobility (μ_e_) of the EOD was determined to be 1.01 × 10^−4^ cm^2^ V^−1^ s^−1^. For the HODs, the pristine film exhibits a sluggish hole mobility (μ_h_ ≈ 2.50 × 10^−5^ cm^2^ V^−1^ s^−1^). Considering its large injection barrier, the pristine device is largely injection-limited, making it highly hole-deficient. However, upon employing the CA-41 HIL, the hole mobility effectively surges to 9.50 × 10^−5^ cm^2^ V^−1^ s^−1^. Rather than representing an absolute equality in the emissive layer, this magnitude indicates that the hole supply capability of the HIL is finally elevated to the same order of magnitude as the electron transport. This crucial supply matching mitigates the anode-side hole bottleneck, promoting more balanced carrier injection and reducing interfacial carrier accumulation. Consequently, non-radiative losses are minimized, leading to the observed enhancement in radiative recombination efficiency [52].

Crucially, the CA-58 film exhibits a nominally higher apparent hole mobility of 1.24 × 10^−4^ cm^2^ V^−1^ s^−1^ and an unusually low turn-on voltage (3.53 V), presenting a physical paradox given its 0.53 eV secondary barrier. This discrepancy is attributed to morphology-induced inhomogeneity rather than uniform bulk transport. At this extreme concentration, excessive depletion of the insulating PSS binder leads to severe PEDOT aggregation, creating localized conductive pathways where the effective barrier is lowered due to field concentration. Consequently, while the average interface impedes hole injection (leading to accumulation), these localized pathways facilitate high current flow, resulting in a mismatch between average energetics and local transport. This severe carrier injection imbalance causes surplus holes to accumulate at the interface, triggering exciton–polaron quenching. This mechanism aligns with recent findings that excessive hole accumulation, whether from leakage or imbalanced transport, significantly degrades efficiency and stability via exciton-polaron annihilation [8]. In contrast, CA-41 maintains excellent carrier balance, achieving the optimal efficiency.

**Figure 5:**
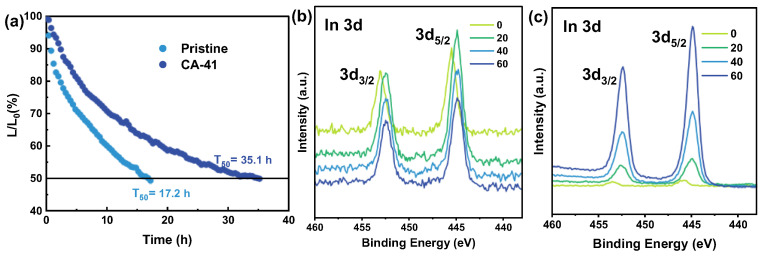
Operational stability and interfacial chemical passivation. (**a**) Normalized luminance decay characteristics (L/L_0_) versus operation time for green OLEDs with pristine and CA-41 PEDOT:PSS HILs, measured at an initial luminance of 1000 cd m^−2^. XPS depth profiling spectra of the In 3d core level for (**b**) the pristine PEDOT:PSS film and (**c**) the optimized CA-41 treated film coated on ITO substrates, tracked at various etching times. The black horizontal line in (**a**) marks L/L_0_ = 50% for determining T_50_.

In addition to the high efficiency, improved operational stability is a fundamental prerequisite for the practical application of OLEDs. Conventional PEDOT:PSS HILs suffer from strong intrinsic acidity and hygroscopicity. These characteristics induce chemical etching of the ITO anode and the subsequent diffusion of indium ions into the organic layer, which drastically shortens the operational lifetime of the devices [53]. To evaluate the impact of the selective reconstruction strategy on device durability, the operational lifetime of the optimized CA-41 device and the pristine reference was continuously monitored at a constant driving current with an initial luminance (L_0_) of 1000 cd m^−2^ (Figure 5).

As depicted in the normalized luminance decay characteristics, the control device based on pristine PEDOT:PSS exhibits rapid luminance degradation, reaching 50% of its initial value (T_50_) after only 17.2 h. Conversely, the CA-41 device demonstrates robust operational stability, maintaining half of its initial luminance for 35.1 h—an impressive 2.0-fold extension in its operational lifespan. This substantial improvement indicates that the optimized citric-acid treatment does not reduce device durability; instead, it significantly enhances operational stability. This behavior is mechanistically ascribed primarily to the chemical neutralization of interfacial acidity, complemented by the reduced content of hygroscopic PSS components. Although the surface remains hydrophilic, the removal of surface PSS chains reduces the density of hygroscopic sulfonate groups. More fundamentally, the CA treatment induces a chemical neutralization via proton exchange, mitigating the acidic corrosion of the ITO anode and suppressing indium ion migration, which is crucial for long-term device stability [54]. This anti-corrosion effect is unambiguously confirmed by XPS depth profiling analysis. To investigate the elemental distribution, the films were sequentially etched with an argon ion beam. As shown in Figure 5b, the pristine PEDOT:PSS film exhibits prominent In 3d peaks (In 3d_5/2_ and In 3d_3/2_) even at the top surface and upper bulk regions (etch times of 0 s and 20 s), indicating severe Indium diffusion driven by the chemical etching of the underlying ITO anode by the highly acidic PSS chains. In contrast, the CA-treated films show significantly suppressed In signals at the interface. This direct evidence of mitigated ITO corrosion provides a plausible origin for the approximately twofold enhancement in operational lifetime.

Conversely, the CA-41-treated film (Figure 5c) shows no detectable Indium signal in the corresponding upper layers. The In 3d peaks only emerge after prolonged etching (≥20 s) when the ion beam reaches the ITO interface. This notable compositional contrast proves that the CA-mediated proton exchange effectively eradicates the interfacial acidity. By preserving the chemical integrity of the anode interface and preventing heavy-metal ion poisoning of the organic emissive stack, the CA-41 HIL ensures sustained and stable charge injection throughout prolonged operation.

Compared with conventional PEDOT:PSS modification strategies that mainly emphasize conductivity enhancement, the present CA-mediated treatment highlights the need to balance conductivity, work-function adjustment, interfacial acidity, morphology, and ITO stability for OLED applications. However, the treatment window is concentration-dependent: excessive CA treatment can cause an over-shifted work function and a rougher, more aggregated PEDOT-rich morphology, which lowers the overall efficiency of CA-58 despite its high lateral conductivity. Future work will therefore focus on refining the treatment window, evaluating long-term stability under broader operating conditions, and extending this mild surface-reconstruction strategy to other emissive systems and solution-processed OLED architectures.

## 4. Conclusions

In summary, we demonstrated a mild citric-acid treatment strategy for selectively reconstructing the PEDOT:PSS surface in Ir(ppy)_3_-based phosphorescent OLEDs. The optimized CA-41 treatment improved the electrical conductivity of the HIL, adjusted the work function for more favorable energy-level alignment, reduced interfacial acidity, and stabilized the ITO/PEDOT:PSS interface. XPS depth profiling confirmed that the optimized treatment suppressed indium diffusion from ITO, supporting the role of interfacial chemical stabilization in enhancing operational stability. As a result, the optimized device achieved a maximum EQE of 20.37% and an approximately twofold increase in operational lifetime. These results indicate that weak-acid-driven PEDOT:PSS surface reconstruction is an effective strategy for improving both efficiency and stability in PEDOT:PSS-based phosphorescent OLEDs.

## Figures and Tables

**Table 1:** The summary of the detailed performance parameters of devices with different CA-treated HILs.

HIL Type	Von [V]	Max. CE [cd A^−1^]	Max. EQE [%]	Max. PE [lm W^−1^]
Pristine	3.76	58.90	16.83	24.97
CA-24	3.69	62.38	18.02	30.21
CA-41	3.56	70.20	20.37	42.18
CA-58	3.53	54.79	15.46	23.64

Von is defined as the voltage at a luminance of 1 cd m^−2^. CE, PE, and EQE denote current efficiency, power efficiency, and external quantum efficiency, respectively.

## Data Availability

The data presented in this study are available on request from the corresponding author.

## Supplementary Materials

The following supporting information can be downloaded at https://www.mdpi.com/article/10.3390/polym18091104/s1, Figure S1: OLED device architecture; Figure S2: Molecular structures of key materials; Figure S3: Water contact angle image of the CA-24-treated PEDOT:PSS film; Table S1: Film thicknesses of PEDOT:PSS films under different citric acid (CA) treatment conditions, measured by a ET150 surface profilometer (Kosaka, Japan).

## Abbreviations

The following abbreviations are used in this manuscript:

HIL	Hole injection layer
OLEDs	Organic light-emitting diodes
PEDOT:PSS	Poly(3,4-ethylenedioxythiophene):poly(styrene sulfonate)
CA	Citric acid
XPS	X-ray Photoelectron Spectroscopy
AFM	Atomic Force Microscopy
UV-vis	Ultraviolet–visible spectroscopy
EL	Electroluminescence
CE	Current Efficiency
PE	Power Efficiency
EQE	External Quantum Efficiency
HODs	Hole-only devices
EODs	Electron-only devices
SCLC	Space-charge limited current
