# Supplementary material for: Citric Acid-Treated PEDOT:PSS with Optimized Interfacial Energetics for Phosphorescent OLEDs Achieving over 20% EQE and Extended Lifetime
Source: Polymers (Basel). 2026 Apr 30;18(9):1104. doi: 10.3390/polym18091104 (PMC13165924; doi:10.3390/polym18091104)
Supplement: Supplementary file 1 [file polymers-18-01104-s001.zip › polymers-4270721-supplementary.pdf]

Supplementary Materials

Citric Acid-Treated PEDOT:PSS with Optimized Interfacial Energetics for Phosphorescent OLEDs Achieving Over 20% EQE and Extended Lifetime

Ming Wu[1,2], Wenqing Zhu[1,2,*], Zhiyin Feng[1,2], Qidi Lin[1,2], Huang Lu[1]

[1] School of Materials Science and Engineering, Shanghai University, 99 Shangda Road, Shanghai 200444, China

[2] Key Laboratory of Advanced Display and System Applications, Ministry of Education, Shanghai University, 149 Yanchang Road, Shanghai 200072, China

*Correspondence: wqzhu@shu.edu.cn

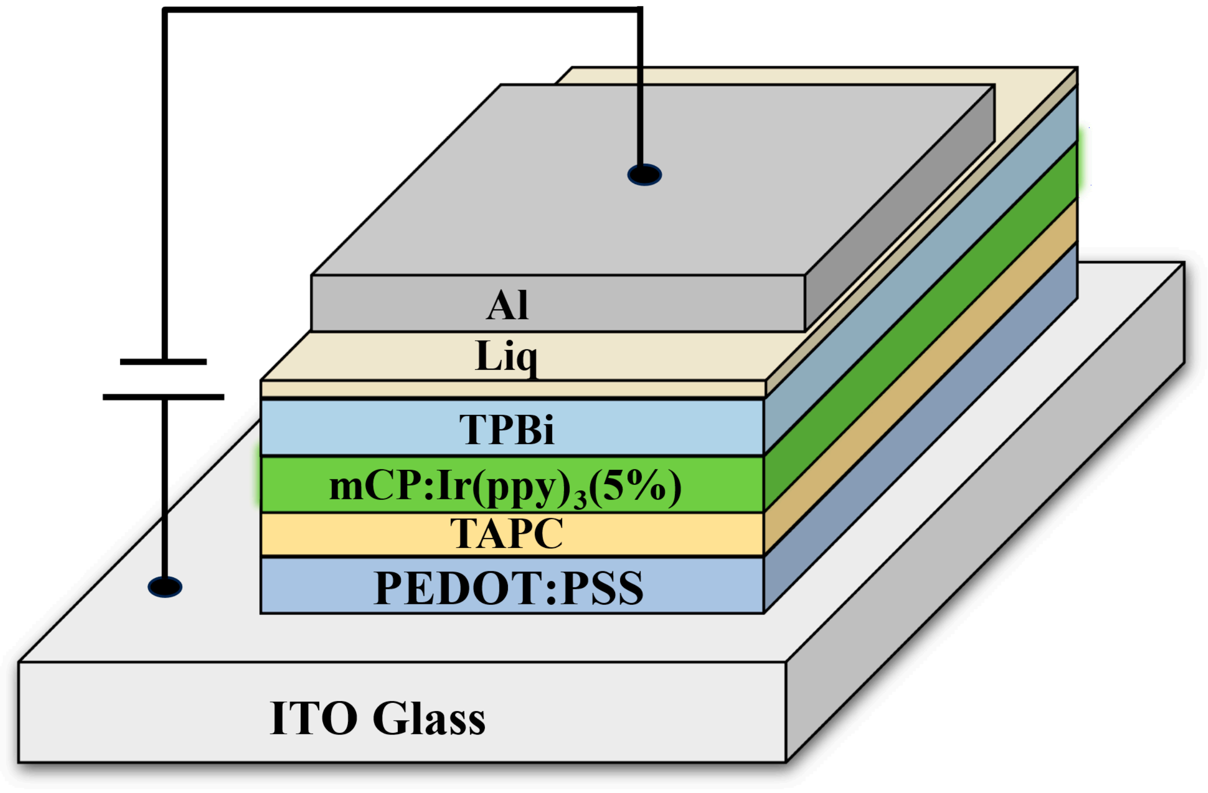

Figure S1. OLED device architecture.

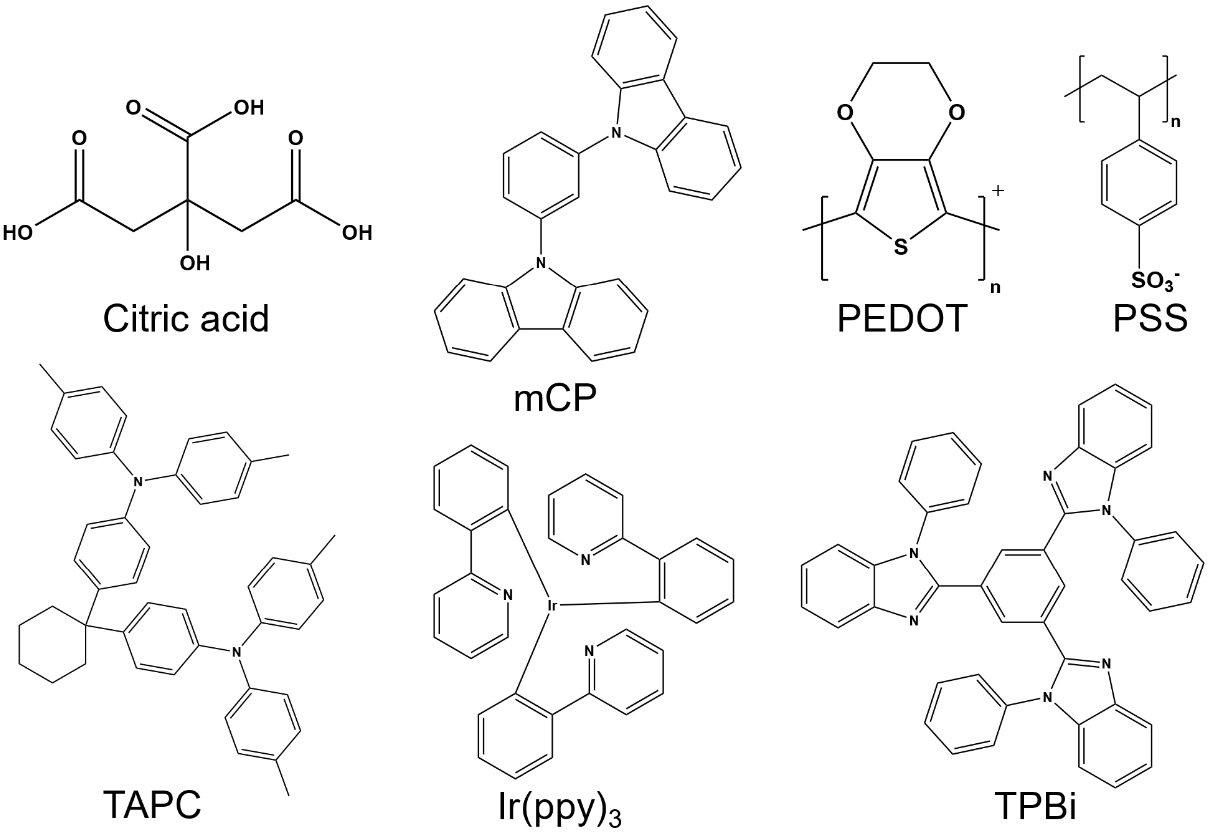

Figure S2. Molecular structures of key materials.

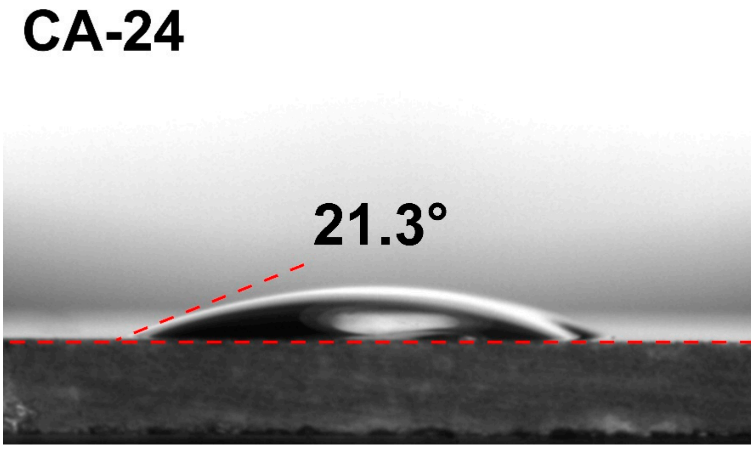

Figure S3. Water contact angle image of the CA-24-treated PEDOT:PSS film.

Table S1. Film thicknesses of PEDOT:PSS films under different citric acid (CA) treatment conditions, measured by a ET150 surface profilometer (Kosaka, Japan).

Sample	Thickness (nm)	Standard Deviation (nm)	Number of Measurements
Pristine	35.3	±0.3	5
CA-24	29.6	±0.2	5
CA-41	29.9	±0.2	5
CA-58	26.3	±0.3	5
